# Inhibition of miRNA-34a Promotes M2 Macrophage Polarization and Improves LPS-Induced Lung Injury by Targeting Klf4

**DOI:** 10.3390/genes11090966

**Published:** 2020-08-20

**Authors:** Mohd Junaid Khan, Prithvi Singh, Ravins Dohare, Rishabh Jha, Arshad H. Rahmani, Saleh A. Almatroodi, Shakir Ali, Mansoor Ali Syed

**Affiliations:** 1 Translational Research Lab, Department of Biotechnology, Faculty of Natural Sciences, Jamia Millia Islamia, New Delhi 110025, India; mohd.khanj@gmail.com; 2 Centre for Interdisciplinary Research in Basic Sciences, Jamia Millia Islamia, New Delhi 110025, India; prithvi.mastermind@gmail.com (P.S.); ravinsdohare@gmail.com (R.D.); rishabhjha.bioinfo@gmail.com (R.J.); 3 Department of Medical Laboratories, College of Applied Medical Sciences, Qassim University, Buraydah 51452, Saudi Arabia; ah.rahmani@qu.edu.sa (A.H.R.); smtrody@qu.edu.sa (S.A.A.); 4 Department of Biochemistry, School of Chemical and Life Sciences Jamia Hamdard, New Delhi 110025, India; sali@jamiahamdard.ac.in

**Keywords:** macrophage subsets, microRNAs, acute lung injury, innate immunity

## Abstract

Acute respiratory distress syndrome (ARDS) is an outcome of an accelerated immune response that starts initially as a defensive measure, however, due to non-canonical signaling, it later proves to be fatal not only to the affected tissue but to the whole organ system. microRNAs are known for playing a decisive role in regulating the expression of genes involved in diverse functions such as lung development, repair, and inflammation. In-silico analyses of clinical data and microRNA databases predicted a probable interaction between miRNA-34a (miR-34a), mitogen-activated protein kinase 1 (ERK), and kruppel like factor 4 (Klf4). Parallel to in silico results, here, we show that intra-tracheal instillation of lipopolysaccharides (LPS) to mice enhanced miR-34a expression in lung macrophages. Inhibition of miR-34a significantly improved lung histology, whereas over-expression of miR-34a worsened the lung injury phenotype. miR-34a over-expression in macrophages were also demonstrated to favour pro-inflammatory M1 phenotype and inhibition of M2 polarization. In a quest to confirm this likely interaction, expression profiles of Klf4 as the putative target were analyzed in different macrophage polarizing conditions. Klf4 expression was found to be prominent in the miR-34a inhibitor-treated group but down-regulated in the miR-34a mimic treated group. Immuno-histopathological analyses of lung tissue from the mice treated with miR-34a inhibitor also showed reduced inflammatory M1 markers as well as enhanced cell proliferation. The present study indicates that miR-34a intensified LPS-induced lung injury and inflammation by regulating Klf4 and macrophage polarization, which may serve as a potential therapeutic target for acute lung injury/ARDS.

## 1. Introduction

Acute respiratory distress syndrome (ARDS) is inflammatory disorder that is characterized by respiratory failures resulting in considerable mortality rates worldwide [1]. ARDS is an outcome of both direct and indirect insults, with aspiration, inhalation of toxic gas, diffuse pulmonary infection, near-drowning, and lung contusion being the direct ones, whereas sepsis, non-thoracic trauma, hyper transfusion, and cardiopulmonary bypass are the indirect insults [2,3,4]. ARDS is characterized by pulmonary oedema resulting due to disintegration of the epithelial–endothelial barrier, leading to increased permeability, neutrophil migration, and an influx of pro-inflammatory mediators in interstitial space, thus resulting in compromised gas exchanges and lung functions [1,5]. Macrophages play a significant part in innate immune responses, progression and resolution of ARDS, and related inflammatory conditions [6,7,8]. Macrophage polarization is a well-established phenomenon wherein macrophages exist in different phenotypes corresponding to its polarized states, which is purely dependent upon effectors and external cues [9,10,11,12,13].

miRNAs are short nucleotide sequences ranging from 20–25 nt in length and originating mainly from non-coding genomic loci as pri-miRNAs containing a stem-loop structure of around 1 kb in size by RNA polymerase II enzyme, which is later processed by DROSHA and DICER enzymes in nucleus and cytoplasm, respectively, to give rise to mature miRNA [14,15]. miRNAs are established as regulators of various inflammatory genes in both innate and adaptive immune cells [16]. Expression profiles of different miRNAs are changed in different pathophysiological states of the lungs [17,18,19,20]. Variable expressions of miRNAs under a different set of conditions also influence the macrophages to polarize to pro-inflammatory or anti-inflammatory states, thus either aggravating the state of inflammation or alleviating it [10]. Amongst the different effectors, kruppel like factor 4 (Klf4) is one of the prominent ones that modulates macrophages to polarize to anti-inflammatory M2 phenotype known for resolution of inflammation and tissue repair [21]. Klf4 is a transcription factor with nuclear localization, which is conserved evolutionarily and is characterized conformationally as zinc fingers. Klf4 is known to regulate the expression of a gene involved in diverse cellular processes that are context-dependent, such as cell growth arrest, cell survival, cell differentiation, and resolution of inflammation [22,23,24,25,26].

miR-34a is known to be induced in different lung injury models in both in-vivo and in-vitro models [27,28,29]. The upregulated miR-34a leads to the activation of inflammation-associated genes, thereby promoting the pro-inflammatory state of macrophages [27,30,31]. Lipopolysaccharides (LPS), a toll-like receptor 4 (TLR4) ligand, is a well-known regulator of mitogen-activated protein kinase/extra-cellular-regulated kinase (MAPK/ERK) signalling, which is marked by the secretion of several pro-inflammatory mediators [32,33]. ERK inhibition studies have shown downward trends in pro-inflammatory response, even if cells were treated by LPS [34,35]. Thus, we hypothesize LPS-induced miR-34a targets Klf4, which is a proven target of miR-34a [36], promotes pro-inflammatory M1 phenotype, and worsens acute lung injury (ALI) in coordination with MAPK/ERK; therefore, inhibition of miR-34a would be beneficial in terms of ALI and macrophage polarization status.

The pulmonary phenotypes of ALI (Intra-tracheal LPS) mice model or ARDS patients are characterized by Patchy intra-alveolar polymorphonuclear (PMN) cells infiltrate and change endothelial permeability [37]. Given the possible role of miRs in the pathogenesis of ALI or ARDS, in this study, the differentially expressed genes profile of ARDS/sepsis patients was downloaded and analyzed. Through differential expression analysis, we identified regulatory miRNAs, including miR-34a and transcription factors. We found enhanced lung miR-34a expression in ALI mice models and over-expression of miR-34a worsened lung phenotype. Inhibition of miR-34a in macrophages limited inflammation regulating polarization toward M2 and improved pulmonary phenotype. We also show the role of Klf4, one of the downstream targets of miR-34a, in macrophage polarization and inflammation. Together, our findings support miR-34a as a novel therapeutic target in regulating LPS-induced acute lung injury.

## 2. Materials and Methods

### 2.1. Differentially Expressed Genes (DEGs) Screening from the mRNA Expression Profile

The National Center for Biotechnology Information (NCBI)-Gene Expression Omnibus (GEO) database (http://www.ncbi.nlm.nih.gov/geo) [38] was searched for ARDS-associated mRNA expression profile selection. The GEO2R (https://www.ncbi.nlm.nih.gov/geo/geo2r/) web tools were accessed for doing the R-based analysis of GEO datasets to identify the differentially expressed genes (DEGs) between two groups, i.e., sepsis only and sepsis with acute lung injury. Genes having a *p*-value < 0.05 were considered as the DEGs. These DEGs corresponding to ${log}_{2}\left(Fold\;Change\right)>0$ and ${log}_{2}\left(Fold\;Change\right)<0$ were categorized as up and downregulated, respectively. The protein–protein interaction (PPI) of the screened DEGs was created using an online tool, STRING v11.0 (https://string-db.org/) [39], by selecting interaction pairs corresponding to an overall score > 0.9 Cytoscape v3.7.2 [40] was used to construct and subsequently visualize the PPI network. Top 10 DEGs were ranked based on each of the six specific centrality measures, i.e., maximal clique centrality (MCC), edge percolated component (EPC), closeness, betweenness, bottleneck, and radiality, using CytoHubba plugin available in Cytoscape.

### 2.2. Significant miRNAs and Transcription Factors (TFs) Retrieval for Feed-Forward Loop (FFL) Construction

The list of TFs regulating hub genes was extracted from ChIPBase v2.3 [41] and ChEA v3.0 [42] databases, respectively. Total TFs obtained from both databases were searched exhaustively via available literature studies, and the ones having an association with ARDS/ALI were considered as the final TFs. miRNAs interacting with our hub genes and TFs were fetched from miRWalk v3.0 [43] and StarBase v2.0 [44] databases, respectively. A two-tier screening process was implemented for extracting the miRNAs from miRWalk where, in the first tier, parameters such as binding gap = 1, binding region = 3UTR, and score ≥ 0.95 were set as the preferred threshold criteria. The first-tier filtered miRNAs that were common in all the three source databases of miRWalk, i.e., TargetScan, miRDB, and miRTarBase, were selected in the second-tier. All these interaction pairs were then merged to form a three-node miRNA feed-forward loop (FFL) and were visualized using Cytoscape.

### 2.3. Materials

LPS (L4524, lipopolysaccharides from *Escherichia coli* 055: B5) was obtained from Sigma (St. Louis, MO, USA). The following antibodies, namely, mouse monoclonal anti-KLF4, anti-beta Actin, rabbit polyclonal anti-Arg1, and mouse monoclonal anti-II1beta were purchased from Santa Cruz Biotechnology (Santa Cruz, CA, USA), and rat polyclonal anti-NOS2 was purchased from Bio-legend (San Diego, CA, USA). Goat anti-mouse and anti-rabbit Horse readdish peroxidase (HRP) conjugated secondary antibodies were purchased from Santa Cruz Biotechnology, and goat anti-rat HRP conjugate was purchased from Bio-Legend. Hematoxylin and eosin stains were purchased from Merck. miR-34a inhibitor and miR-34a mimics were synthesized from Qiagen (Hilden, Germany).

Bradford Protein estimation reagent, First-strand cDNA synthesis kit, and Syber Green Master mix were purchased from Biorad (Hercules, CA, USA). TRIzol reagent and Radioimmunoprecipitation (RIPA) buffer were purchased from Thermo Fisher Scientific (Waltham, MA, USA).

### 2.4. Murine ALI Model

Six to eight weeks old pathogen-free C57BL6 mice male or female weighing 18–25 g in a group of four were used in this study. The animals were housed in a temperature and humidity regulated environment with proper light/day (L/D) cycles and provided food and water ad libitum. The study was ethically approved and conducted as per the guidelines of Jamia Hamdard animal ethics committee (file number: 1358) and *Committee for Control and Supervision of Experiments on Animals (CPCSEA), Govt. of India*. Mice were injected with ketamine (ketamine 100 mg/kg) and xylazine (10 mg/kg) intraperitoneally. LPS at a dose of 10 mcg per mice was administered intratracheally. For the treatment groups, 50 µL of sterile phosphate-buffered saline (PBS) with miR-34a mimic and miR-34a inhibitor at doses of 2 mg/kg body weight were given intraperitoneally.

### 2.5. Cell Culture and Transient Transfection

Murine macrophage RAW264.7 cells were purchased from National Centre for Cell Sciences (NCCS, Pune, India) and cultured in Dulbecco’s Modified Eagles’ medium (Gibco, Carlsbad, CA, USA) supplemented with 10% Fetal Bovine Serum (Gibco) and 1% antibiotic-antimycotic (Gibco) in a humidified chamber saturated with 5% CO_2_ at 37 degrees Celsius of optimum temperature. miR-34a mimics, anti-miRNA-34a, and scrambled oligos obtained from Qiagen were transfected at 30 picomolar using lipofectamine (Invitrogen, Carlsbad, CA, USA) for 16 h, and then cells were treated after 24 h.

### 2.6. Macrophage Polarization

RAW264.7 cells were treated with LPS at a dose of 100 nanograms/mL to obtain M1 phenotypes macrophages and with interleukin 4 (IL4) at a dose of 20 ng/mL to obtain M2 polarized macrophages. Representative M2 markers used for this study were *Arg-1*, *Ym1*, *Fizz1, Klf4,* and *β-actin* with the following sequences, *Arg-1*, forward: 5′CAGAAGAATGGAAGAGTCAG3′, reverse: 5′CAGATAGTCAGGGAGTCACC3′, *Fizz1*, forward: 5′GCTGATGGTCCCAGTGAATAC3′, reverse: 5′CCAGTAGCAGTCATCCCAGC3′, YM1, forward: 5′GCAGAAGCTCTCCAGAAGCAATCCTG3′, reverse: 5′ATTGGCCTGTCCTTAGCCCAACTG3′, *Klf4*, forward: 5′ACTTGTGACTATGCAGGCTG3′,reverse: 5′ACACTTCTGGCACTGAAAGG3′, *β-actin*: 5′CTGTCCCGCTATGCCTCTG3′, reverse: 5′ATGTCACGCACGATTTCC3′.

### 2.7. Histopathological Analyses

Lung tissues were harvested from mice post euthanasia and were fixed in 10% neutral buffered formalin solution (Sigma). Then, the formalin-fixed tissues were embedded in paraffin and sectioned into 5 µm thick sections. Later, it was subjected to the standard procedure of deparaffination along with rehydration in continuation with antigen retrieval. Then, subsequent probing by Klf4, Ki67, interleukin 1 beta (IL1β), and myeloperoxidase (MPO) (Santa Cruz Biotechnology) antibodies with corresponding HRP conjugated secondary antibodies (Santa Cruz Biotechnology). 3.3’-Diaminobenzidine (DAB) stating procedure was used for immune-histolochemical development. Later, slides were analyzed under a light microscope (MEIJI, Saitama, Japan) and quantified by Image J software, vs. 1.8.0_111.

### 2.8. Semiquantitative PCR

Total RNA was extracted from the lung tissue by TRIzol reagent (Invitrogen) as per the instruction of the manufacturer. cDNA library of the mRNAs from the transcriptome was prepared by iScript cDNA synthesis kit (Bio-Rad, Hercules, CA, USA). Primers for target gene and marker genes were obtained from IDT (Iowa, IA, USA) and Eurofins.

### 2.9. miRNA Quantification

Total RNA was extracted from cell lines or lung tissues using TRIzol reagent (Invitrogen); then, cDNA was synthesized using miscript cDNA synthesis kit (Qiagen). Syber green dye-based miRNA quantitation kit (Qiagen) and real-time PCR instrument ABI Step One plus (Applied Biosystem, Foster, CA, USA) were used to perform the reaction. The ct values were used for further quantifications. U6 Small nuclear RNA was used as an endogenous control miRNA expression analysis.

### 2.10. Western Blot Analysis

Total protein was extracted from RIPA lysis buffer and quantified by Bradford assay. The extracted protein was allowed to separate on 12% SDS-PAGE and was later transferred to a Polyvinylidene (PVDF) membrane (Bio-Rad). Sequential probing by targeted primary antibody followed by HRP conjugated secondary antibody was done with intermittent washing by Tris-buffered Saline with 1% Tween-20 (TBST). Then, chemiluminescence was observed by adding enhanced chemiluminescence (ECL) (Bio-Rad) substrate to the PVDF membrane. The following antibodies were used: KLF4, IL1β, β-actin (1:1000 dilutions; Santa Cruz Biotechnology), inducible nitic oxide synthase (iNOS), Arginase-1 (1:1000: Biolegend), and, correspondingly, HRP-conjugated secondary antibodies (1:10,000 dilutions; Santa Cruz Biotechnology) were used as per the host specificity.

### 2.11. Statistical Analyses

Statistical significance of the quantified data was done by t-Test and two-way ANOVA following Tukey’s post hoc analyses. Values are presented as mean +/− SD. *p*-value < 0.05 was considered as significant.

## 3. Results

### 3.1. ALI-Associated DEGs, miRNAs, and Hub Gene(s) Prediction

**Figure 1:**
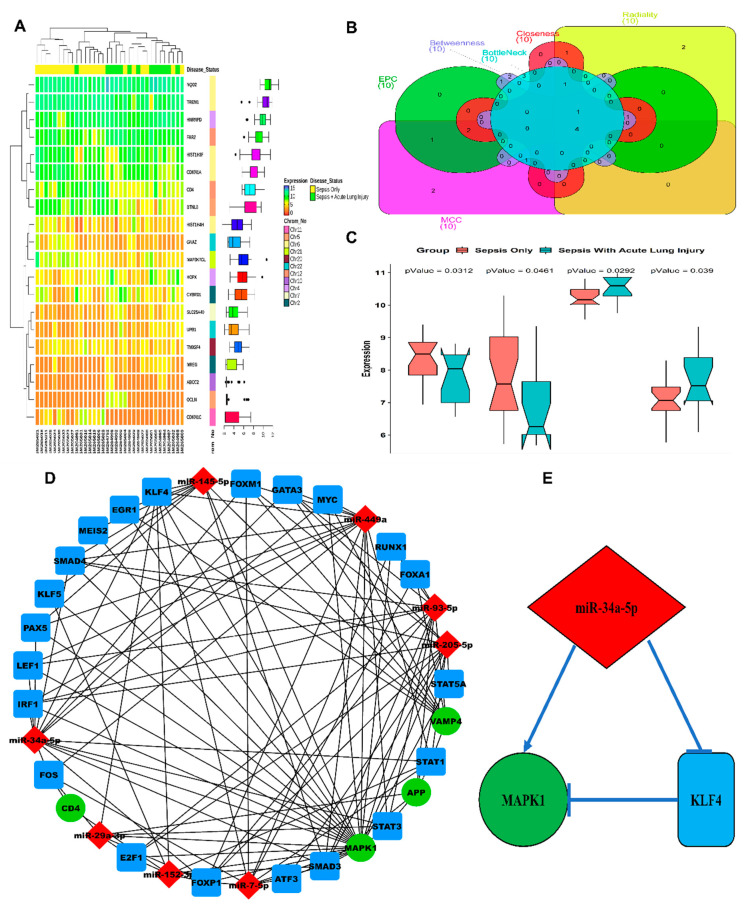
In silico analysis of differentially expressed genes (DEGs) and miRNAs in acute lung injury patients. (**A**) Heatmap plot of top 10 up and down-regulated DEGs. The expression values for each DEG (row) are normalized across all the samples (columns). Hierarchical clustering using Pearson metric and complete linkage method were used for categorizing the data into two disease status groups, i.e., sepsis only and sepsis with acute lung injury. Cluster dendrograms for row and column are displayed along the left and the top sides of the plot. The location of each DEG on its respective chromosome is shown in the right as the row annotation bar. The boxplots in the right panel display the expression data distribution density of each DEG. The line inside each boxplot represents the median, whereas the endpoints of the axis are labelled by minimum and maximum values. (**B)** Venn plot for identifying the significant hub genes from the protein–protein interaction (PPI) network. Areas with varying colours correspond to different centralities (maximal clique centrality, i.e., MCC, edge percolated component, i.e., EPC, closeness, betweenness, bottleneck, and radiality). Top 10 ranked PPI DEGs based on each of the six centrality measures are shown in the Venn plot by different gene set colours, and their intersectional area reveals the four hub genes. (**C**) Notched boxplots comparing the relative expression levels of our hub genes (*App*, *Cd4*, *Mapk1*, *Vamp4*) in two different groups, i.e., sepsis only and sepsis with acute lung injury. Significance for aggregated expression values was determined by the Wilcoxon *p*-values. The notches signify a 95% confidence interval for medians. (**D**) Graphical plot of a three-node miRNA feed-forward loop (FFL) regulatory network. The nodes in green signify our hub genes, nodes in red and blue signifiy the acute lung injury (ALI)/acute respiratory distress syndrome (ARDS)-linked miRNAs and transcription factors (TFs), respectively. (**E**) The higher-order subnetwork comprising one miRNA (miR-34a-5p), TF (Klf4), and our hub gene (*Mapk1*) along with their relative regulatory interaction type.

Following the search criteria specified, we selected the dataset with accession number GSE10474 from GEO. This dataset comprised a total of 34 patient samples (13 patients with ALI + sepsis and 21 patients with sepsis only). A total of 197 DEGs were filtered based on the threshold (*p*-value < 0.05). Additionally, 69 downregulated and 128 upregulated DEGs were categorized corresponding to the ${log}_{2}\left(Fold\;Change\right)$ threshold. A list of all DEGs is available in Supplementary Table S1. An annotation heatmap of the top 10 up and downregulated DEGs is in Figure 1A. The centrality-based PPI network analysis revealed four hub genes, i.e., *App*, *Cd4*, *Mapk1*, and *Vamp4* (Figure 1B). Notched-boxplots comparing the relative gene expression levels of our hub genes are shown in Figure 1C.

Three regulatory interaction pairs (miRNA-gene, TF-gene, miRNA-TF) were formed to construct the three-node miRNA FFL, which consisted of a total of 33 unique nodes and 114 edges (Figure 1D). Among these edges, 14 belonged to miRNA-gene links, 27 belonged to TF-gene links, and 73 belonged to miRNA-TF links. Additionally, among the 33 nodes, four belonged to our hub genes, 21 belonged to human ARDS-linked TFs, and eight belonged to human ARDS-linked miRNAs. The top higher-order subnetwork (based on the degree) in our FFL consisted of one TF (Klf4), one hub gene (*Mapk1*), and one miRNA (miR-34a-5p) (Figure 1E).

A total of eight ARDS-linked miRNAs targeted our four hub genes. *Mapk1* was targeted/repressed by all the eight extracted miRNAs. miR-93-5p targeted the largest number of hub genes (i.e., three). The maximum number of miRNAs targets top three TFs were forkhead box P1 (Foxp1), smad family member 3 (Smad3), and Klf4, respectively. Both miR-34a-5p and miR-449a repressed the largest number of TFs (i.e., 13, Figure 1D). A total of 21 TFs regulated our hub genes where *Mapk1* was regulated by a maximum number of TFs (i.e., 13, Figure 1D).

### 3.2. LPS Induced mir-34a in Lung Macrophages Accompanied with the Severity of Lung Injury

**Figure 2:**
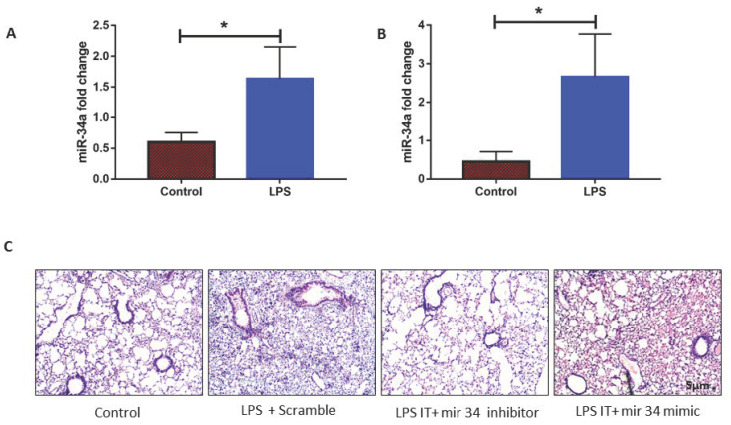
Lipopolysaccharides (LPS) induced miR-34a induction increases lung injury. (**A**) miR-34a expression was induced in mice lung after 24 h of intra-tracheal administration (10 mcg/mice) of LPS. (**B**) Treatment of LPS in RAW264.7 cell line also induced miR-34a expression after 16 h. (**C**) Mice were treated with LPS along with miR-34a inhibitor, mimics, and scrambled, lung tissues were stained with Hematoxyline & Eosine, and histology was seen in the light microscope. Test of significance was done by Student t-test. * *p* < 0.05, (four animals were used).

miR-34a expression was found to be notable in the conditions of ARDS/ALI as per our in-silico data analysis, and its role in the regulation of lung injury in different models has been shown by us and others [45,46,47]. Hence, to ascertain the role of miR-34a in ALI, we detected miR-34a expression in mouse lungs and RAW macrophages and found enhanced expression of miR34a in macrophages after LPS induction. Similarly, compared to the control group, the LPS instilled mice group showed a considerable increase in the expression of miR-34a as compared to the control group. To check the specific effect of miR-34a expression on lung phenotype, we gave miR-34a inhibitor or miR-34a mimic via the intraperitoneal route. Then, 50 µL (2 mg/kg body weight) of miR-34a inhibitor or mimic (or scrambled control) was administered intraperitoneally, along with LPS instillation via the trachea. Lung histology displayed that, in comparison to the scrambled group, intraperitoneal administration with miR-34a inhibitor in mice significantly improved the LPS induced ALI phenotype compared to scrambled, specifically in terms of intra-alveolar PMN infiltration and patchy areas (Figure 2C). However, administration of miR-34a mimics further deteriorated lung phenotype as compared to scrambled control (Figure 2C).

**Figure 3:**
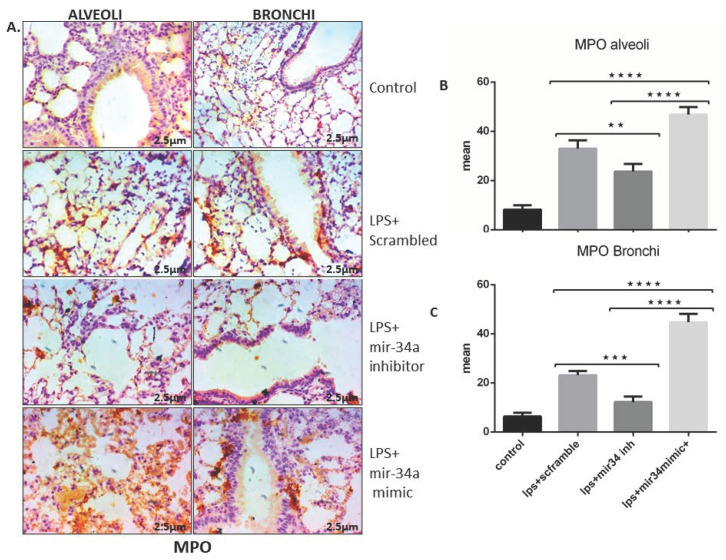
miR-34 inhibition reduced expression of LPS induced myeloperoxidase (MPO) in mice. (**A**) IHC stained representative lung slides shows the expression of MPO in alveolar and bronchial sections of lung tissue in different treatment groups. (**B**,**C**) expression of MPO quantified and statistically analyzed using two-way ANOVA *** *p* < 0.001, **** *p* < 0.0001 (*n* = 4).

Administration of miR-34a inhibitor was also effectively reduced lung inflammation, as evident by diminished MPO activity in lung intra-alveolar and bronchus area of ALI (IT) mice (Figure 3A–C). Contrary to this, intra-peritoneal administration of miR-34a mimics enhanced MPO staining in intra-alveolar and bronchial areas of LPS (IT) lung sections (Figure 3A–C), showing the inflammatory nature of miR-34a.

### 3.3. miR-34a Expression Downregulates Klf4 Expression in Macrophages

**Figure 4:**
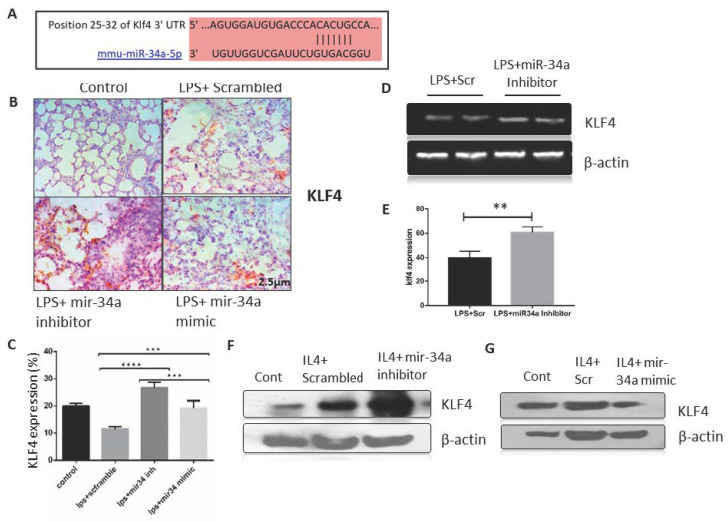
mir-34a targets KLF4 in lung macrophages. (**A**) Mir-34a-5p aligned with *Klf4* to depict the putative binding site corresponding to ‘3’ untranslated region (UTR) region of *Klf4* mRNA. (**B**) IHC staining of Klf4 in lungs of different treatment groups was done, and (**C**) counting of positive cells was performed to determine the expression of Klf4 in mice lung tissue and was analyzed by two-way ANOVA following Tukey’s post hoc analyses (**D**,**E**). RT-PCR results for the gene Klf4 from the lung tissue on 1.5% agarose gel and its densitometry by normalizing against actin. Statistical significance was done by t-test. (**F**,**G**) Immunoblots of Klf4 from RAW264.7 macrophage transfected with miR-34a inhibitor or mimic. ** *p* < 0.01, *** *p* < 0.001, **** *p* < 0.0001 (a minimum of four animals were used for each study).

To identify the specific molecular targets of miR-34a, we used in silico approach for predicting molecular targets using FFL construction from ARDS/sepsis patients data (Figure 1D) and three source databases of miRWalk, i.e., TargetScan, miRDB, and miRTarBase. We ended up with Klf4 as a potential target of miR-34a, which was confirmed by the sequence complementarity between miR-34a-5p and ‘3’ untranslated region (UTR) of *Klf4* mRNA (Figure 4A.). *Klf4* signaling proved to be a critical player in lung injury and inflammation, and several studies have also shown its role in macrophage polarization [36,48,49]. Patterns of *Klf4* expression were observed in different treatment groups, both in animal ALI models and in macrophages, as shown in Figure 4B,C. *Klf4* expression was decreased by roughly 50–60% in LPS-induced lungs as compared to saline controls. Administration of miR-34a inhibitor significantly induced KLF4 immunostaining, specifically in intra-alveolar spaces, whereas miR-34a mimic further reduced klf4 expression in LPS (IT) induced lungs (Figure 4B). Moreover, quantification of IHC staining by image j also proved a similar trend with statistically significance values (Figure 4C). RT-PCR data also showed augmentation of *Klf4* expression upon treatment of miR-34a inhibitor in LPS (IT) lungs as compared to controls (Figure 4D,E). We further confirmed these effects on macrophages by transfecting them with miR-34a inhibitor or mimic or scrambled and stimulating them with M2 polarizing IL-4. We found IL-4 stimulated Klf4 protein expression was further enhanced in the miR-34a inhibitor-treated group, although miR-34a mimic-treated cells showed diminished *Klf4* expression as compared to scrambled control in macrophages (Figure 4F,G).

### 3.4. miR-34a Regulates Macrophage Polarization

**Figure 5:**
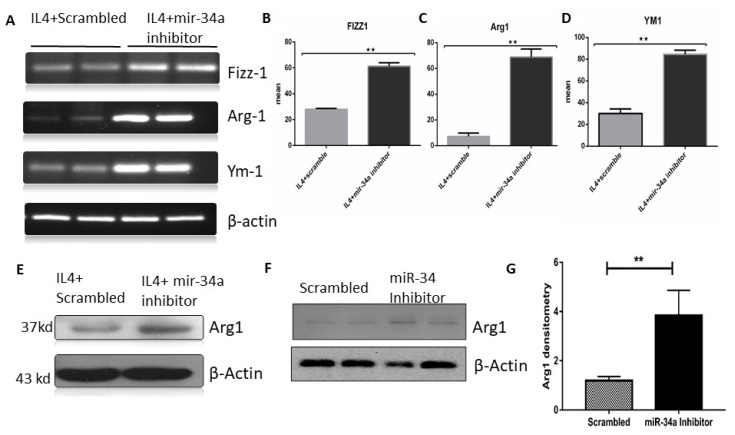
miR-34a inhibition promoted M2 phenotype and inhibited M1 markers. RAW264.7 cells were transfected with miR-34a scrambled or inhibitor or mimic and treated with IL-4 for 16 hrs. (**A**) RT-PCR was performed for M2 phenotype marker genes *Klf4*, *Arg-1*, Fizz-1, and Ym1. (**B**–**D**) Gel bands were quantified by densitometry and normalized by actin. (**E**–**G**) Immunoblot of Arg1 in scrambled and miR-34a inhibitor-treated groups with or without IL-4. Statistical difference between the groups analyzed by t-Test. ** *p* < 0.01 (*n* = 4).

Based on our hypothesis, next, we asked whether the change in miR-34a expression in macrophages was responsible for these effects on the lungs. We transfected RAW cells with miR-34a inhibitor or scrambled and polarized towards M2 using IL-4; representative markers, namely, *Agr-1*, *Fizz1*, and Ym1, showed higher expression in the miR-34a inhibitor transfected group as compared to the scrambled control (Figure 5A). Furthermore, we confirmed, using densitometric analysis, that all markers showed significant enhancement of M2 markers mRNA expression in the miR-34a inhibitor transfected groups (Figure 5B–D). Furthermore, AGR-1 (anterior gradient-1) protein expression was also enhanced in the miR-34a inhibitor transfected group as compared to the scrambled control after IL-4 stimulation in RAW macrophages (Figure 5E). Even transfection of miR-34 inhibitor alone was sufficient to stimulate Arg1 in RAW macrophages (Figure 5F,G).

**Figure 6:**
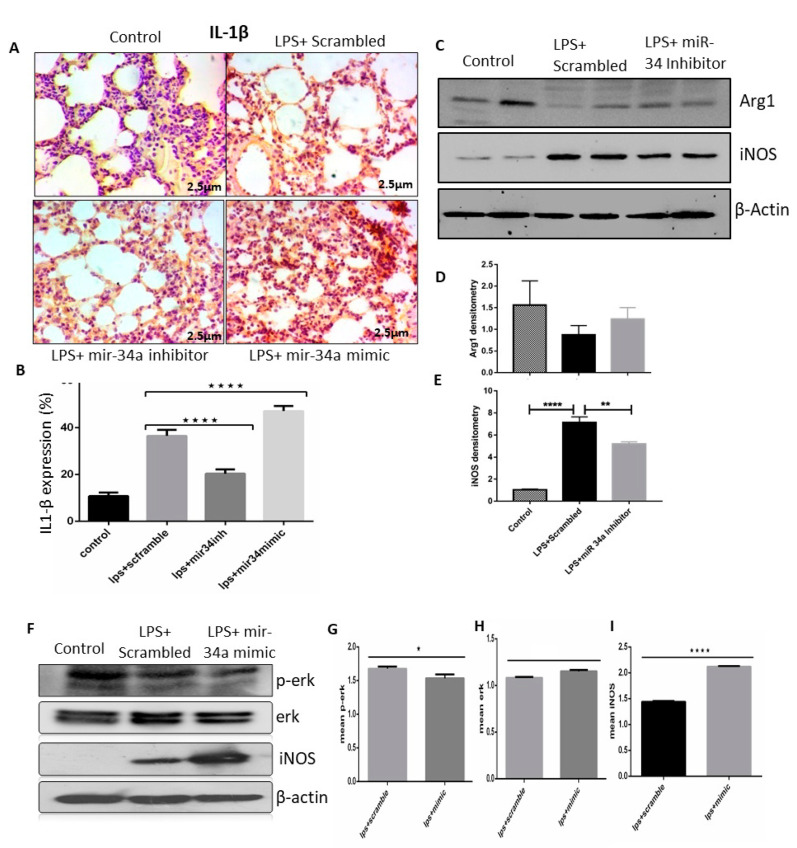
LPS effectively induced miR-34a, which promoted the M1 phenotype of macrophages. (**A**) Lung IHC staining of control, LPS + scrambled, and miR-34a + LPS treated mice groups showed expression of IL1-β. (**B**) Color density of IL1-β was quantified and analyzed using image J and two-way ANOVA following Tukey’s post hoc analyses. (**C**–**E**) Immunoblots of Arg1 and iNOS were performed in lung tissue lysates of mentioned groups. Densitometry was performed for all immunoblots and analyzed by two-way ANOVA following Tukey’s post hoc analyses. (**F**–**I**) RAW macrophages transfected with miR-34a inhibitor or scrambled; after treatment with LPS, cell lysates were performed to check the expression of ERK, p-ERK, and iNOS in given groups. Densitometry was done, and t-tests were used for analysis. * *p* < 0.05, ** *p* < 0.01, **** *p* < 0.0001 (*n* = 4).

To check the involvement of miR-34a on LPS induce M1 polarization and lung injury, we first checked the IL-1β M1 marker in lung tissue of LPS (IT) animals with miR-34a inhibitor or mimic treatment. As expected, miR-34a mimic treatment enhanced lungs IL-1β expression as compared to LPS (IT) control group, although treatment of miR-34a inhibitor significantly reduced IL-1β positive cells (Figure 6A,B). Treatment of miR-34a inhibitor also inhibited LPS induced iNOS, which is also one of the markers of M1 polarized macrophages, in mice lung tissues as compared to scrambled treated group. Expression of M2 specific marker Arg1 was also enhanced in the miR-34a inhibitor-treated group as compared to the control (Figure 6C,D). However, iNOS was enhanced in the LPS+miR-34a mimic transfected group as compared to the LPS+ scrambled in RAW macrophages (Figure 6E,F). Transfection of miR-34a mimic also enhanced LPS and induced nitric oxide secretion as compared to scrambled control, but miR-34a transfection did not show any effect (Supplementary Figure S1A,B). The involvement of the *Erk1/2*ERK1/2 pathway regulated LPS stimulated inflammatory cytokines production [50,51,52], and here we found that transfection of miR-34a inhibitor reduced LPS induced *Erk* phosphorylation, although miR-34a mimic transfection did not exert any notable effect on *Erk* phosphorylation (Figure 6F–H). Results obtained were significant enough to say that miR-34a is a pro-inflammatory microRNA enhancing the M1 polarized state of macrophages.

### 3.5. miR-34a Augments the Effects of LPS Arresting Cell Proliferation

**Figure 7:**
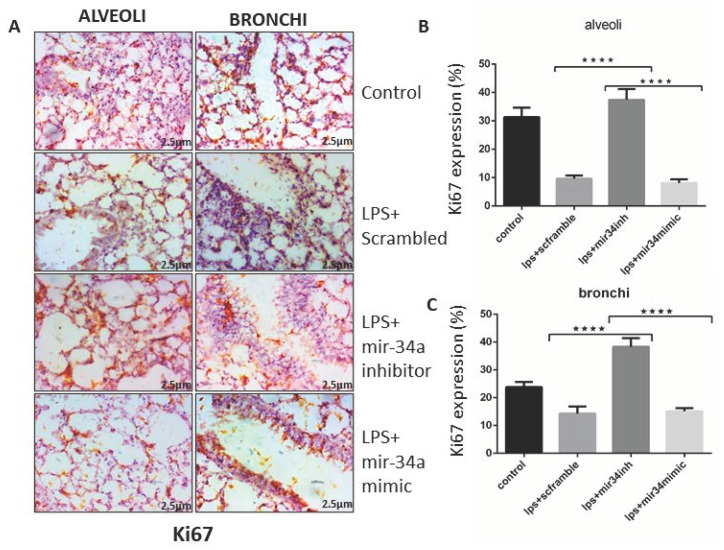
miR-34a reduced cell proliferation. (**A**) Immune-histological images of mice lung tissue showed the expression of Ki67 after treatment with LPS along with miR-34a inhibitor, mimics, and scrambled treated groups. (**B**,**C**) Expression of Ki67 quantified in alveolar and bronchial areas and statistically analyzed by two-way ANOVA. **** *p* < 0.0001 (*n* = 4).

LPS is not only known for being pro-inflammatory, but it also slows down the rate of cell proliferation [44]. To examine the potential of miR-34a inhibition on lung cells regenerative capacity in mice, these were stained with Ki67, a prominent marker of cell proliferation. miR-34a, which gets upregulated by LPS treatment, reduced Ki67 staining and almost arrested the proliferation of cells, which was effectively visible in the IHC slides of lung tissue of both alveolar and bronchial region, whereas Ki67 appeared to be less expressed in LPS and LPS+mir-34a mimic groups. However, in the LPS+ miR-34a inhibitor group, it showed more expression of Ki67 as compared to the control (LPS+scrambled) lungs (Figure 7A–C).

## 4. Discussion

Our study reports three major findings. First, LPS induced miR-34a expression in lung macrophages of mice. Second, inhibition of miR-34a demonstrated a protective function in LPS induced ALI phenotype, regulating inflammatory and cell proliferation pathways. Third, we experimentally validated the downstream target, Klf4, and showed its role in macrophage polarization and lung injury.

Several studies have found that miRNAs can regulate inflammatory response by modulating immune mediators at the post-transcriptional level, suggesting miRs play a critical role in innate immune response in ALI [53,54]. Findings based on in-silico, in vitro, and in vivo data displayed that the expression of miR-34a was up-regulated in the lung injury models. Our results in this study were consistent with these reports, and we found that the level of miR-34a increased in the lungs of ALI (IT) mice models and LPS-induced macrophages. To the best of our knowledge, this is a unique study that connected the miR-34a–KLF4 axis to inflammatory lung injury. Hence, we detected that inhibition of miR-34a improved lung phenotype of LPS (IT) ALI mice models, showing that miR-34a could be involved in the process of LPS-induced ALI. However, research shows that the absence of miR-34a could alleviate the inflammatory response [55,56].

We initialized this study with in-silico analyses of a clinical data set of ALI/sepsis cases from GEO datasets to access proteins coded by DEGs and also did the miRNA-gene prediction using miR walk v3.0 to capitalize on hub genes and transcription factors. miRNA FFL network analysis showed a probable interaction between miR-34a, *Mapk1*, and Klf4. Interestingly, miR-34a was notably upregulated in several studies in the ALI, and its interaction with Klf4 and MAPK/ERK was already demonstrated by numerous studies [36,57].

Another study on miR-34a also suggested induction in LPS-TLR4 signalling as well as its role in pro-inflammatory iNOS production [27,58]. Neutrophil infiltration is one of the outcomes of lung inflammation shown by myeloperoxidase (MPO) expression in affected lungs [59,60,61]. As expected, we also observed the same wherein MPO expression was heightened in alveolar and bronchial spaces of lungs in LPS (IT)+scrambled group, which was further enhanced with concomitant mir-34a mimic administration in LPS (IT) mice lungs, suggesting the role of miR-34a in endothelial dysfunction in lungs [62,63].

Angiopoietin (*Ang1*), cyclin-dependent kinases (*CDKs*), TEK receptor tyrosine kinase (*Tie-2*), and BCL2 apoptosis regulator (*Bcl2)* are well-established targets of miR-34a, which have important roles in inflammation and lung injury [29,45]. To identify the mechanism behind the protective effects of miR-34a, we performed in-silico analysis of miRNA databases to identify targets and recognized few related to inflammation/macrophage polarization. Finally, we focused on *Klf4* because we and others have proven it has a significant role in lung injury and inflammation [26,64,65]. *Klf4* is a proven modulator of M2 macrophage polarization and is an experimentally recognized putative miR-34a target in several studies [36,65,66], and it was downregulated in our miR-34a over-expressed tissues and macrophages. In vivo, *Klf4* was significantly increased by miR-34a antagonism in LPS induced lung injury, and this signalling was able to ameliorate the acute lung injury phenotype. This *Klf4* expression was responsible for augmenting polarization of macrophages toward the M2 phenotype. However, miR-34a over-expression significantly reduced *Klf4* in lung and macrophages. Similar to our results, other studies also connected *Klf4* expression and inflammation, which has shown beneficial effects in resolution of lung injury and inflammation [26,64]; however, few contrary studies show the role of *Klf4* in promoting iNOS expression and hyper-inflammation [67]. Our data thus suggest a critical role of miR-34a, the downstream *KLF4* signalling, and the transition between M1 and M2 macrophage phenotypes, which is supposed to be important for the molecular regulation of functional shaping of macrophages in the associated ALI phenotype. We thus propose miR-34a-Klf4 signalling as the major contributor to miR-34a-mediated lung injury.

We also showed LPS-induced miR-34a is a potent pro-inflammatory microRNA that promotes M1 polarization markers, specifically IL1-β and iNOS. The role of nitric oxide signalling is well established in lung development and injury [52]. We also observed enhanced LPS-induced phosphorylation of ERK in miR-34a over-expression treated groups further enhanced LPS-induced p-ERK in macrophages. Additionally, we observed modulation of phosphorylation of ERK after miR-34a inhibitor treatment. ERK is a transcription factor that migrates to nucleus to activate the expression of inflammatory genes, and several studies have shown the role of ERK signalling in miR-34a mediated inflammatory response and lung injury [45]. Although the dose of LPS considered in this particular study was sufficient enough to evoke an immune response, higher and lethal doses of LPS [68] would certainly allow us to understand the mechanism of onset and the resolution of inflammation in a more effective way.

Other studies have specified that miR-34a reduces cell proliferation and invasion. Parallel with those studies, we also detected that miR-34a expressed more in LPS (IT) lung, and cell proliferation was notably reduced. Furthermore, miR-34a inhibitor had the reverse effect, signifying that miR-34a can significantly affect cellular proliferation. miR-34a is known to be a tumour suppressor gene that promotes senescence and cell death, thereby inhibiting cell cycle and cell proliferation [69,70]. In our study, we got LPS-induced abrogation of cell proliferation, indicated by Ki67 immunostaining, in miR-34a over-expressed lung tissues that were significantly improved after miR34a inhibitor treatment. The effects of miR-34a mediated cell cycle regulation and cell proliferation have been shown in various studies specifically targeting different CDK’s, such as CDK1, CDK6 [70,71].

However, we also acknowledge the limitations of our study. First of all, our results were based on the expression of miR-34a expression in macrophages only, although the lung consists of several other types of cells. More studies, such as miR-34a expression in epithelial or endothelial cells and other acute lung injury mouse models, are needed to prove its functionality. Another limitation is that we did not further validate the direct Klf4–miR-34a interactions. However, some of the previous studies already proved miR-34a–Klf4 mRNA interactions [26,64]. Future research on miR-34a and its targets in macrophages will be informative for authenticating its functions in ARDS and will provide a more comprehensive understanding of mechanistic knowledge.

## 5. Conclusions

In conclusion, miR-34a exacerbates LPS-induced ALI by targeting *Klf4* in mice model and macrophages. Beyond this observation, improvement in ALI and resolution of inflammation are key affirmations by miR-34a antagomir treatment. miR-34a directly targets *Klf4*, and macrophage polarization was also confirmed, overexpressing miR-34a in lung and macrophages. Overall, we can speculate that miR-34a inhibition augments *Klf4*, which polarizes macrophages toward the M2 phenotype, which might help to resolve inflammation and ALI. miR-34a might be a perfect candidate to regulate inflammation and ALI. Further research on the miR-34a expression and the downstream Klf4 might provide a wider approach and method for the diagnosis and the treatment of ALI caused by sepsis and lung injury.

## Supplementary Materials

The following are available online at https://www.mdpi.com/2073-4425/11/9/966/s1, Figure S1: (A) NO secretion of transfected RAW264.7 cells after LPS stimulation, (B) NO secretion of transfected RAW264.7 cells after IL-4 treatment, Table S1: List of all differentially expressed genes (DEGs).
