# Supplementary material for: Inhibition of miRNA-34a Promotes M2 Macrophage Polarization and Improves LPS-Induced Lung Injury by Targeting Klf4
Source: Genes (Basel). 2020 Aug 20;11(9):966. doi: 10.3390/genes11090966 (PMC7563942; doi:10.3390/genes11090966)
Supplement: Supplementary file 1 [file genes-11-00966-s001.zip › Supplementary Table S1_.pdf]

Probe ID	Gene symbol	P.Value	logFC
213348_at	CDKN1C	0.004151512	-1.643876923
220421_at	BTNL8	0.008235855	-1.46422381
202284_s_at	CDKN1A	0.000234726	-1.318039194
206110_at	HIST1H3F	0.03799973	-1.135337729
219434_at	TREM1	0.013971354	-1.089658608
208180_s_at	HIST1H4H	0.036565506	-1.066553114
221211_s_at	MAP3K7CL	0.034916617	-1.060173626
212194_s_at	TM9SF4	0.003390107	-0.995667399
204993_at	GNAZ	0.031691653	-0.982175458
203547_at	CD4	0.046114034	-0.935738462
218791_s_at	KATNBL1	0.043899165	-0.933693773
206206_at	CD180	0.033972199	-0.905068864
203305_at	F13A1	0.048883375	-0.851722344
221853_s_at	NOMO1	0.034472975	-0.815011722
212099_at	RHOB	0.006429499	-0.801652747
211136_s_at	CLPTM1	0.021519816	-0.78942381
208161_s_at	ABCC3	0.045311945	-0.782235897
210009_s_at	GOSR2	0.016943859	-0.771656777
218611_at	IER5	0.044637972	-0.765412088
212647_at	RRAS	0.022521168	-0.752482418
201236_s_at	BTG2	0.001911733	-0.738475458
200825_s_at	HYOU1	0.035329404	-0.728552747
201360_at	CST3	0.049446279	-0.693534432
204494_s_at	C15orf39	0.049745312	-0.670304029
209619_at	CD74	0.038532325	-0.64843956
218175_at	CCDC92	0.041520512	-0.646552381
212322_at	SGPL1	0.043392345	-0.641659707
218059_at	ZNF706	0.025574447	-0.628916117
200742_s_at	TPP1	0.030532379	-0.626882418
201841_s_at	HSPB1	0.04189037	-0.625118681
222043_at	CLU	0.043877426	-0.621557509
212415_at	SEPT6'	0.028127783	-0.585452747
200602_at	APP	0.031269617	-0.567405128
205367_at	SH2B2	0.04530079	-0.562387546
211936_at	HSPA5	0.024862171	-0.555584982
222231_s_at	LRRC59	0.046521912	-0.555267399
216396_s_at	EI24	0.048216946	-0.553284982
36564_at	RNF19B	0.029594557	-0.551598168
217775_s_at	RDH11	0.039261059	-0.5509663
214730_s_at	GLG1	0.045741381	-0.535169231
208836_at	ATP1B3	0.022855504	-0.530912088
204367_at	SP2	0.028458032	-0.521842857
212038_s_at	VDAC1	0.031185771	-0.509422711
215343_at	CCDC88C	0.040054571	-0.506532967
214749_s_at	ARMCX6	0.023970713	-0.506371795
221620_s_at	APOO	0.02418586	-0.500782418
218321_x_at	STYXL1	0.014217856	-0.487591575
200859_x_at	FLNA	0.049938901	-0.47185641
203557_s_at	PCBD1	0.035956485	-0.449632967

32032_at	TSSK2	0.016323636	-0.423063004
200734_s_at	ARF3	0.003453177	-0.417653846
209699_x_at	AKR1C2	0.034373202	-0.409137363
216457_s_at	SF3A1	0.042472813	-0.404283883
201500_s_at	PPP1R11	0.002912154	-0.392455311
203740_at	MPHOSPH6	0.042972736	-0.388342857
204925_at	CTNS	0.032125981	-0.386678022
52164_at	C11orf24	0.047991296	-0.356412821
209606_at	CYTIP	0.019665145	-0.352827839
202116_at	DPF2	0.043550543	-0.322546154
218753_at	XKR8	0.008067966	-0.30708022
209044_x_at	SF3B4	0.042648775	-0.301903297
201961_s_at	RNF41	0.024240533	-0.297467766
209158_s_at	CYTH2	0.033347902	-0.289932234
202767_at	ACP2	0.016159487	-0.272072161
201692_at	SIGMAR1	0.019009806	-0.266516117
211072_x_at	TUBA1B	0.029315704	-0.23743956
211750_x_at	TUBA1C	0.047135404	-0.227974359
208648_at	VCP	0.044784255	-0.224466667
218601_at	URGCP	0.030623676	-0.149813919
210288_at	KLRG1	0.044966395	0.015390476
209993_at	ABCB1	0.03011227	0.033857875
214441_at	STX6	0.023698395	0.035866667
221031_s_at	APOLD1	0.032288838	0.037842125
219347_at	NUDT15	0.043521825	0.039857143
201815_s_at	TBC1D5	0.012013741	0.041811722
220148_at	ALDH8A1	0.049443433	0.045769597
222238_s_at	POLM	0.047900196	0.055085714
208157_at	SIM2	0.049299019	0.06045641
216206_x_at	MAP2K7	0.044521194	0.06418022
205219_s_at	GALK2	0.040522763	0.064949817
209524_at	HDGFRP3	0.029727559	0.066935897
212849_at	AXIN1	0.025988048	0.070994139
215412_x_at	PMS2P8	0.046556384	0.074452381
211536_x_at	MAP3K7	0.045201962	0.092866667
216439_at	TNK2	0.033686157	0.113465201
222241_at	MVB12B	0.032098211	0.114192674
214699_x_at	WIPI2	0.044427272	0.115385348
210100_s_at	ABCA2	0.02041355	0.115893407
220391_at	ZBTB3	0.034276797	0.120190476
208102_s_at	PSD	0.037504486	0.135445421
210474_s_at	CDK11A	0.003690227	0.153143223
205050_s_at	MAPK8IP2	0.049532767	0.158653114
214873_at	LRP5L	0.01132581	0.163721245
215994_x_at	TBC1D9B	0.028846256	0.165278388
45653_at	KCTD13	0.003214014	0.176134066
213681_at	CYHR1	0.023811399	0.181169597
210768_x_at	TMCO1	0.041336039	0.191095971
204531_s_at	BRCA1	0.029072901	0.202239927
201246_s_at	OTUB1	0.021600177	0.203750916

222131_x_at	RHOT2	0.006232498	0.226112821
209957_s_at	NPPA	0.03118378	0.227171062
207988_s_at	ARPC2	0.035372357	0.23614652
218977_s_at	TRNAU1AP	0.040850787	0.236854579
212005_at	SZRD1	0.012715704	0.245955678
202401_s_at	SRF	0.046796243	0.249187912
211513_s_at	OGFR	0.049397668	0.250412821
210758_at	PSIP1	0.028798175	0.254132967
202009_at	TWF2	0.046791168	0.258027106
219401_at	XYLT2	0.007694277	0.25931978
207805_s_at	PSMD9	0.048234162	0.260878022
212786_at	CLEC16A	0.012347648	0.261174359
203035_s_at	PIAS3	0.031716278	0.263417216
204959_at	MNDA	0.008641128	0.264717949
209364_at	BAD	0.047547667	0.265711722
201233_at	PSMD13	0.032254554	0.269031136
220143_x_at	LUC7L	0.037984021	0.275419048
35265_at	FXR2	0.019846292	0.276971062
213042_s_at	ATP2A3	0.045612075	0.280827839
221190_s_at	C18orf8	0.030870999	0.283559707
218274_s_at	ANKZF1	0.041335582	0.287065201
206593_s_at	MED22	0.015256795	0.291707326
48825_at	ING4	0.034026166	0.295404762
201230_s_at	ARIH2	0.021185041	0.317619414
212271_at	MAPK1	0.029258976	0.32024359
209735_at	ABCG2	0.044696182	0.323361905
209390_at	TSC1	0.037729922	0.324241392
213204_at	CUL9	0.032610091	0.326230403
209539_at	ARHGEF6	0.039228152	0.329265568
221922_at	GPSM2	0.046440168	0.330608791
206855_s_at	HYAL2	0.038837499	0.338400366
206077_at	KEL	0.013627532	0.345363736
219822_at	MTRF1	0.045113598	0.346177289
213444_at	ZNF862	0.040951288	0.352456777
219065_s_at	DPY30	0.044748571	0.355247253
222059_at	ZNF335	0.024221635	0.366076557
218969_at	PAM16	0.036223102	0.368998901
212034_s_at	EXOC7	0.013133118	0.371837363
213403_at	MFSD9	0.019259779	0.37351685
205944_s_at	CLTCL1	0.046466542	0.374608425
212892_at	ZNF282	0.035231091	0.387061172
212147_at	SMG5	0.03522959	0.388089744
214186_s_at	HCG26	0.03617016	0.391948352
218795_at	ACP6	0.022638907	0.396721978
202978_s_at	CREBZF	0.025247909	0.402884982
212181_s_at	NUDT4	0.04689705	0.404234066
203459_s_at	VPS16	0.045810398	0.407087912
205300_s_at	SNRNP35	0.041466331	0.410431502
212475_at	AVL9	0.046996319	0.421823077
204136_at	COL7A1	0.020645687	0.425145788

204605_at	CGRRF1	0.023656385	0.431894139
221769_at	SPSB3	0.00921825	0.454258608
203670_at	ARPC4	0.014183988	0.457925641
201707_at	PEX19	0.030126528	0.461358608
209019_s_at	PINK1	0.021380852	0.468017949
205329_s_at	SNX4	0.048594537	0.469075824
219129_s_at	SAP30L	0.037681799	0.471195604
211935_at	ARL6IP1	0.034797425	0.477020513
212819_at	ASB1	0.00328548	0.478055678
212705_x_at	PNPLA2	0.002258639	0.482990842
210640_s_at	GPER1	0.044983224	0.484584249
202348_s_at	TOR1A	0.029182092	0.48644652
218822_s_at	LOC100652930	0.001057141	0.488689011
218463_s_at	MUS81	0.008262629	0.499675092
202250_s_at	DCAF8	0.032444089	0.505499634
212886_at	CCDC69	0.030122994	0.505764103
202815_s_at	HEXIM1	0.006359182	0.506419414
205839_s_at	TSPOAP1	0.005186267	0.525810989
203467_at	PMM1	0.012153809	0.527440293
213062_at	NTAN1	0.045498007	0.528652015
203273_s_at	TUSC2	0.002842095	0.546394872
213480_at	VAMP4	0.039050388	0.562277656
204833_at	ATG12	0.018747181	0.572030403
221675_s_at	CHPT1	0.030763973	0.574384615
219236_at	PAQR6	0.037152836	0.590370696
219116_s_at	DCUN1D2	0.034660082	0.613138462
214152_at	CCPG1	0.045493148	0.623245421
210140_at	CST7	0.036437045	0.627659341
210328_at	GNMT	0.027016011	0.670813187
201061_s_at	STOM	0.022213094	0.673626374
205577_at	PYGM	0.030413536	0.675141026
209501_at	CDR2	0.044836229	0.697058608
204301_at	KBTBD11	0.031487623	0.713324542
48808_at	DHFR	0.037265856	0.759072161
214606_at	TSPAN2	0.004934591	0.774122344
209799_at	PRKAA1	0.01754624	0.783625641
203336_s_at	ITGB1BP1	0.008850696	0.786734432
219885_at	SLFN12	0.016454884	0.793426374
206155_at	ABCC2	0.013392079	0.8050663
213359_at	HNRNPD	0.022907482	0.82934359
205716_at	SLC25A40	0.028892007	0.87521978
220615_s_at	FAR2	0.015482502	0.910008791
203814_s_at	NQO2	0.002158773	0.91020989
219648_at	MREG	0.029546459	0.922149817
209925_at	OCLN	0.045337798	1.037640293
220507_s_at	UPB1	0.010820493	1.0672
217889_s_at	CYBRD1	0.041109081	1.127380586
211597_s_at	HOPX	0.021836732	1.187104396
