# Supplementary figures and images for: Inhibition of miRNA-34a Promotes M2 Macrophage Polarization and Improves LPS-Induced Lung Injury by Targeting Klf4
Source: Genes (Basel). 2020 Aug 20;11(9):966. doi: 10.3390/genes11090966 (PMC7563942; doi:10.3390/genes11090966)

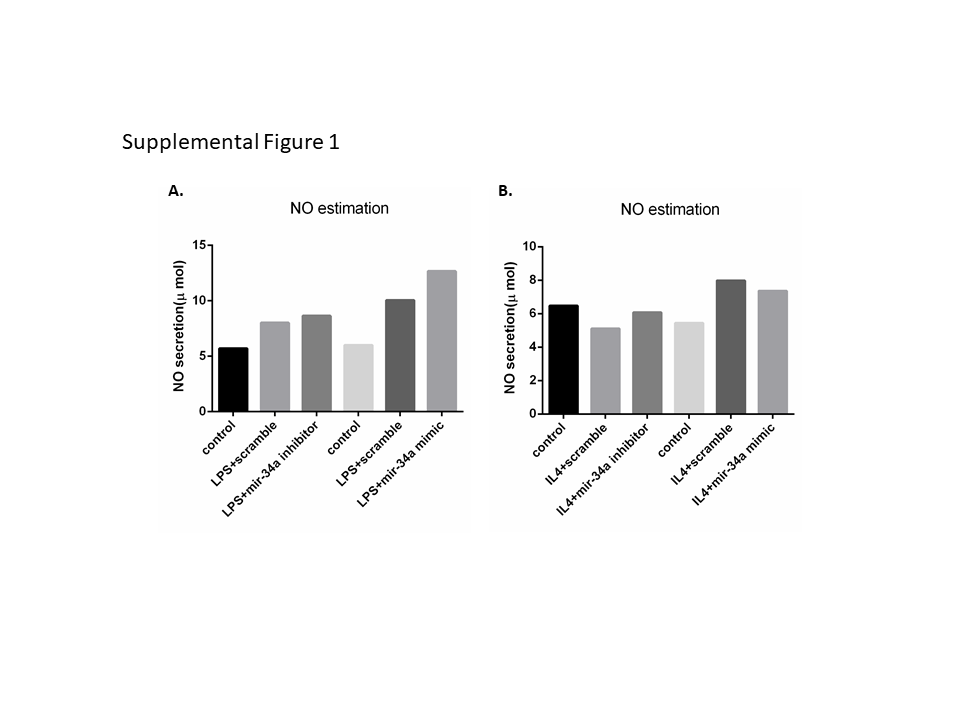

Supplement: Supplementary file 1 [file genes-11-00966-s001.zip › Supplementary Figure S1.png]
